# Synaptic Vesicle Glycoprotein 2C: a role in Parkinson’s disease

**DOI:** 10.3389/fncel.2024.1437144

**Published:** 2024-09-05

**Authors:** Chu Hua Chang, Kah Leong Lim, Jia Nee Foo

**Affiliations:** ^1^Lee Kong Chian School of Medicine, Nanyang Technological University, Singapore, Singapore; ^2^Interdisciplinary Graduate Programme (IGP-Neuroscience), Nanyang Technological University, Singapore, Singapore; ^3^Department of Research, National Neuroscience Institute, Singapore, Singapore; ^4^Genome Institute of Singapore, Agency for Science, Technology and Research (A*STAR), Singapore, Singapore

**Keywords:** synaptic vesicle, Parkinson’s disease, dopamine, synaptic trafficking, Synaptic Vesicle Glycoprotein

## Abstract

Synaptic Vesicle Glycoprotein 2C (SV2C), characterized by its selective expression in discrete brain regions such as the midbrain, has recently emerged as a promising player in Parkinson’s Disease (PD) – a debilitating neurodegenerative disorder affecting millions worldwide. This review aims to consolidate our current understanding of SV2C’s function, its involvement in PD pathogenesis, and to evaluate its potential as a therapeutic target. Integrating previous findings of SV2C, from genetics to molecular studies, and drawing on insights from the largest East Asian genome-wide association study that highlights *SV2C* as a novel risk factor for PD, we explore the potential pathways through which SV2C may influence the disease. Our discussion extends to the implications of SV2C’s role in synaptic vesicle trafficking, neurotransmitter release, and α-synuclein homeostasis, thereby laying the groundwork for future investigations that could pave the way for novel therapeutic strategies in combating PD.

## 1 Introduction

Synaptic vesicles (SVs) are essential in neural communication, packaging and releasing neurotransmitters into the synaptic cleft to facilitate synaptic transmission. The precise regulation of SV loading, transporting and recycling is crucial for maintaining the fidelity of synaptic transmission. Disruptions in this machinery can lead to various neurological disorders, ranging from neurodevelopmental conditions such as Autism Spectrum Disorders to neurodegenerative diseases such as Alzheimer’s Disease and Parkinson’s Disease (PD) (Lepeta et al., 2016).

Within the diverse array of SV proteins, the Synaptic Vesicle Glycoprotein 2 (SV2) family includes three distinct proteins: SV2A, SV2B and SV2C. These integral synaptic membrane proteins play an important role in synaptic processes and comprehensive reviews outlining the structure and proposed functions of SV2 proteins have been detailed by Bartholome et al. (2017), Ciruelas et al. (2019), Hu et al. (2017), and Stout et al. (2019). SV2A is the most extensively-studied paralog due to its implication in epilepsy; it is the target of the anti-epileptic drug levetiracetam (Lynch et al., 2004). Studies on SV2A-deficient mice have revealed its essential role in GABAergic neurotransmission, with the loss of SV2A leading to spontaneous seizures and early post-natal mortality (Crowder et al., 1999). In contrast to SV2A, which is ubiquitous across neuronal synapses, SV2B predominantly localized to excitatory synapses (Bajjalieh et al., 1994). Interestingly, SV2B demonstrates more robust expression in the brain during early development compared to adulthood, although its specific roles in developmental processes remain unclear. While there is some evidence suggesting a potential involvement of SV2B in Alzheimer’s disease, its exact role, particularly in relation to amyloid beta toxicity, remains to be fully elucidated (Detrait et al., 2014). Conversely, our understanding of SV2C remained obscure until recent years. Emerging evidence has begun to unveil its potential involvement in PD, a progressive neurological disorder characterized by the degeneration of dopaminergic neurons in the substantia nigra pars compacta (SNpc) of the midbrain. This association is particularly noteworthy given that SV2C is predominantly expressed in the dopaminergic neurons in the midbrain, though it is also present in GABAergic and cholinergic neurons (Dardou et al., 2013). The majority of PD-linked genes are expressed rather ubiquitously. The exploration of SV2C’s function and its underlying mechanisms within the dopaminergic pathways – which are critically impacted in PD – thus presents an interesting area of research.

This review delves into the current literature surrounding SV2C, particularly its link to PD. It explores the role of SV2C in synaptic neurotransmission and evaluates its potential as a biomarker or therapeutic target. By examining recent studies and pinpointing gaps in our understanding of SV2C’s potential function in PD pathogenesis, this paper aims to highlight the importance of SV2C in neurobiology and its potential significance in advancing PD research.

## 2 Discovery of SV2C and its distinctive localisation in the brain

The functional role of SV2 proteins within the nervous system remains an area of active investigation. SV2 proteins play a crucial role in synaptic transmission, notably in priming SVs for exocytosis and influencing synaptotagmin trafficking (Chang and Südhof, 2009; Yao et al., 2010). They are also recognized as receptors for botulinum neurotoxin A, modulating its entry into the neurons (Dong et al., 2006).

SV2C, the most recently identified member of SV2 protein family, was discovered by Janz and Südhof in 1999. Like its counterparts, SV2A and SV2B, SV2C is characterized by 12 putative transmembrane domains, a cytoplasmic N-terminal tail and a large intraluminal loop (Janz and Südhof, 1999). Each paralog is encoded on a different chromosome – SV2A on chromosome 1, SV2B on chromosome 15 and SV2C on chromosome 5. Despite sharing approximately 80% structural homology with SV2A and SV2B, SV2C’s expression in the brain is notably distinct. Unlike the widespread expression of SV2A and SV2B throughout most regions of the rat brain, SV2C shows restricted expression in phylogenetically older brain regions like the olfactory bulb (OB), the midbrain and the hindbrain. This regional specificity was corroborated by studies in mice and macaque which also demonstrated specific localisation in the striatum and the substantia nigra (Dardou et al., 2011; Dunn et al., 2017, 2019). Intriguingly, these areas are also central to the Braak staging of PD (Braak et al., 2003). Although the exact role of SV2C in PD’s pathological progression is unclear, its selective localisation in these regions hints at a unique and potentially significant role within certain neuronal circuits.

Similar to SV2A, the N-terminal tail of SV2C harbors a unique binding site for synaptotagmin-1 (SYT1), a calcium sensor essential for mediating the fusion of synaptic vesicle membrane with the plasma membrane during neurotransmitter exocytosis (Schivell et al., 2005). Previous investigations revealed that mutation of the endocytosis domain within the amino-terminal SYT1-binding domain of SV2A result in defective trafficking of SYT1 to synaptic vesicles (Haucke and De Camilli, 1999; Yao et al., 2010). While the sequence similarity between SV2A and SV2C suggests a potential role of SV2C in SYT1 trafficking, direct evidence supporting SV2C’s involvement in the trafficking of SYT1 remains elusive.

## 3 Pioneering investigations of SV2C in Parkinson’s disease

The initial link between *SV2C* polymorphisms and PD was reported by Hills-Burns and colleagues, who identified an *SV2C* ortholog in a genome-wide gene–environment interaction study using a nicotine-paraquat *Drosophila* model of PD. Although *SV2C* did not achieve genome-wide significance in their subsequent study, it emerged as the most notable gene. Specifically, single nucleotide polymorphisms (SNPs) within the *SV2C* gene, rs30196 and rs10214163, were identified as having the potential to influence the neuroprotective effects of smoking on PD risk (Hill-Burns et al., 2013). This finding underscores the intricate relationship between genetic factors and environmental exposures, such as tobacco smoking, which has been associated with a reduced PD risk, potentially through the neuroprotective properties of nicotine (Li et al., 2015). Numerous *in vivo* studies have demonstrated that nicotine not only decreases the degeneration of dopaminergic neurons in animal models of PD but also enhances neuronal survival, while concurrently promoting dopamine release (Quik et al., 2008; Turner, 2004). Dunn et al. (2017), suggested that SV2C may mediate nicotine’s modulation of dopamine release, as their study showed that mice lacking SV2C did not exhibit an increase in dopamine release upon nicotine stimulation. Although the relationship between SV2C and nicotine remains unclear, it is possible that SV2C is part of the neurotransmitter pathway modulated by nicotinic acetylcholine receptors on dopaminergic neurons. Nonetheless, the underlying mechanism for this relationship requires further investigation.

Genetic variations within the *SV2C* gene have also been implicated modulating the response to dopamine replacement therapies in PD. Notably, the presence of *SV2C* rs30196 C allele has been correlated with a requirement for lower doses of Levodopa, the primary treatment for managing early-stage PD motor symptoms (Altmann et al., 2016). Conversely, the rs1423099 T allele is associated with a reduced incidence of treatment-related side effects, such as nausea or vomiting (Altmann et al., 2016; Redenšek et al., 2019). While these findings have yet to be replicated in independent studies, such insights may suggest that *SV2C* genetic variants could play a critical role in tailoring PD management strategies, offering a framework for the stratification of PD subtypes and the selection of individualized therapeutic options that maximize efficacy and minimize adverse effects. Given the complexity of genetic influences on treatment responses, further research is essential to fully elucidate the potential of *SV2C* in guiding personalized PD treatment paradigms.

Additionally, it is plausible that these variants, most of which are non-coding, could influence SV2C expression or function. Non-coding variants may affect regulatory elements such as promoters, enhancers, or splice sites, thereby altering the levels of SV2C expression. Changes in SV2C levels could have an effect on synaptic vesicle trafficking and neurotransmitter release. Furthermore, alterations in SV2C function or localization could modulate its interaction with other synaptic proteins. These speculative mechanisms highlight the need for detailed molecular studies to understand the precise impact of SV2C genetic variants on its biological functions and their implications for PD therapy. SV2C is prominently expressed in nearly all dopaminergic neurons within the midbrain, including the substantia nigra and the striatum, pointing to its potential role in modulating dopamine neurotransmission (Dardou et al., 2011). SV2C-deficient mice exhibit elevated gene expression of tyrosine hydroxylase, the rate-limiting enzyme responsible for dopamine synthesis, in the SNpc and displayed diminished locomotor activity in an open field test (Dardou et al., 2013). Notably, the denervation of dopaminergic neurons, induced by MPTP and 6-OHDA lesions, led to a modest but significant upregulation of *SV2C* mRNA in the caudate-putamen on the lesioned side compared to the non-lesioned side using *in situ* hybridization techniques. This suggests SV2C’s involvement in the dopaminergic system, especially under insult. While the exact role of SV2C in dopaminergic neurotransmission and PD pathology remains to be fully elucidated, this study is pivotal in establishing the potential significance of SV2C in dopaminergic neurons and serves as a crucial foundation for future investigations aimed at unraveling the functions of SV2C and its implication for PD.

Dopamine homeostasis is critical for neuronal health, as the accumulation of cytosolic dopamine can increase the vulnerability of dopaminergic neurons (Mosharov et al., 2009). The study by Dunn et al. (2017) identifies a crucial link between SV2C deficiency in mice and disrupted dopamine regulation, manifesting as reduced dopamine content and release, alongside mild motor deficits in locomotion (Dunn et al., 2017). Intriguingly, SV2C was found to co-immunoprecipitate with α-synuclein, although there is currently no evidence for a direct interaction between the two proteins. Furthermore, the presence of increased high molecular weight α-synuclein in SV2C-deficient mice suggests a potential modulatory role of SV2C on the functioning or aggregation processes of α-synuclein. These findings may imply that SV2C may play a crucial role in the pathological changes associated with α-synuclein, potentially influencing its propensity to aggregate. This interaction may underpin key aspects of synaptic dysfunction observed in PD, given the central role of α-synuclein in Lewy body pathology as well as its involvement at presynaptic sites (Calabresi et al., 2023). While the study provides compelling evidence for the role of SV2C in dopaminergic neurotransmission and its possible interaction with α-synuclein aggregation, the use of mouse models necessitates cautious extrapolation to human PD pathology. Future investigations should aim to elucidate the precise mechanisms by which SV2C influences synaptic vesicle dynamics, as well as α-synuclein misfolding or aggregation. This research could potentially illuminate novel therapeutic targets for mitigating synaptic dysfunction or the Lewy body pathology in PD.

## 4 *SV2C* emergence: a novel risk locus for Parkinson’s disease uncovered

The largest East Asian genome-wide association study (GWAS) conducted in 6,724 patients and 24,851 controls identified *SV2C* as a novel risk locus for PD, with the intronic SNP rs246814 showing genome-wide significant association with PD (*p* = 3.48 × 10^−8^; OR = 1.24). This association is replicated across both Asian and European cohorts, with differences in allele frequencies between the two populations (minor allele frequency in Asians: 9–11%; in Europeans: 7–8%) (Foo et al., 2020). This finding was further corroborated by a subsequent study in the European ancestry population by Grover et al. (2021) and observed in the Singapore population by which the variant is present at different frequencies between mild and severe PD subtypes, underscoring SV2C’s potential role in PD risk modulation and disease progression (Deng et al., 2022; Grover et al., 2021). Interestingly, the rs246814 is in complete linkage disequilibrium with the missense variant rs31244, located in exon 10 of *SV2C* gene, which results in the amino acid substitution p.Asp543Asn within SV2C’s luminal domain and could potentially introduce a new N-glycosylation motif (N-X-S/T). The large luminal domain of SV2C protein contains three N-glycosylation sites which are well-conserved across SV2 proteins and binds with high affinity to botulinum neurotoxin A (Dong et al., 2006; Iezzi et al., 2005; Janz and Südhof, 1999). That being said, empirical validation is essential to confirm the impact of this amino acid substitution on SV2C’s N-glycosylation. This highlights a critical gap in our understanding, as the precise role of N-glycosylation alterations in the SV2C’s function and its interaction with other synaptic proteins are yet to be elucidated. Given the crucial role of glycosylation in the luminal loop of SV2 proteins on localizing the protein to the synapse from the endoplasmic reticulum-Golgi apparatus, detailed biochemical and cellular studies are warranted to clarify the biological implications of the rs31244 variant (Nowack et al., 2010). Future investigations should aim not only to validate the functional predictions but also to investigate how glycosylation changes in SV2C might contribute to the pathophysiology of PD, potentially unveiling new disease pathways for therapeutic intervention.

## 5 Advancements and novel perspectives in SV2C—Parkinson’s disease research

Recent investigations have expanded our understanding of SV2C’s functions, particularly its mediation of nicotine effect on autophagy flux, as demonstrated in an α-synuclein-based *Drosophila* model where the effect of nicotine to decrease α-synuclein aggregates were abolished by the knockdown of *SV2C* (Olsen et al., 2023). Autophagy, crucial for degradation and recycling of dysfunctional proteins and organelles, is integral in maintaining protein homeostasis and neuronal health. Dysregulation of this process has been implicated in the pathogenesis of PD, potentially contributing to the synaptic degradation that precedes neuronal loss (Soukup et al., 2018). Whether SV2C influences autophagy, and the precise mechanisms by which it may do so, remain elusive, highlighting the need for future research to dissect its interactions with key players of the autophagic machinery.

In more recent investigations, cutting-edge single-nuclei RNA sequencing analysis on post-mortem substantia nigra from PD patients and healthy controls has revealed a subpopulation of dopaminergic neurons exhibiting a 0.5-fold decrease in *SV2C* expression in the PD cases (Wang et al., 2024). This data aligns with Wu et al.’s (2024) discovery of reduced levels of SV2C in the plasma of PD patients and identified SV2C as a potential biomarker using machine learning algorithms (Wu et al., 2024). The convergence of these findings marks a promising direction for future research, particularly in validating SV2C as a biomarker and clarifying its role in the molecular cascade that characterizes PD progression.

To further investigate the role of SV2C in modulating synaptic vesicle dynamics, recent research found that SV2C promotes the uptake and retention of dopamine in the synaptic vesicles (Bucher et al., 2024). Mice treated with MPTP and deficient in SV2C showed increased vulnerability to the toxin’s effect, suggesting a neuroprotective role for SV2C, potentially by sequestering the toxic compounds from the cytosol. However, no transporter activity has been reported for SV2C till date. It is possible that SV2C mediates its effect of substrate uptake through VMAT2, a monoamine transporter that transports monoamine neurotransmitters into synaptic vesicles. It is intriguing to consider whether SV2C may have a similar effect on cytosolic dopamine, as high levels of cytosolic dopamine have been reported to be neurotoxic (Bucher et al., 2020; Mosharov et al., 2009).

## 6 Discussion and future directions

**Figure 1:**
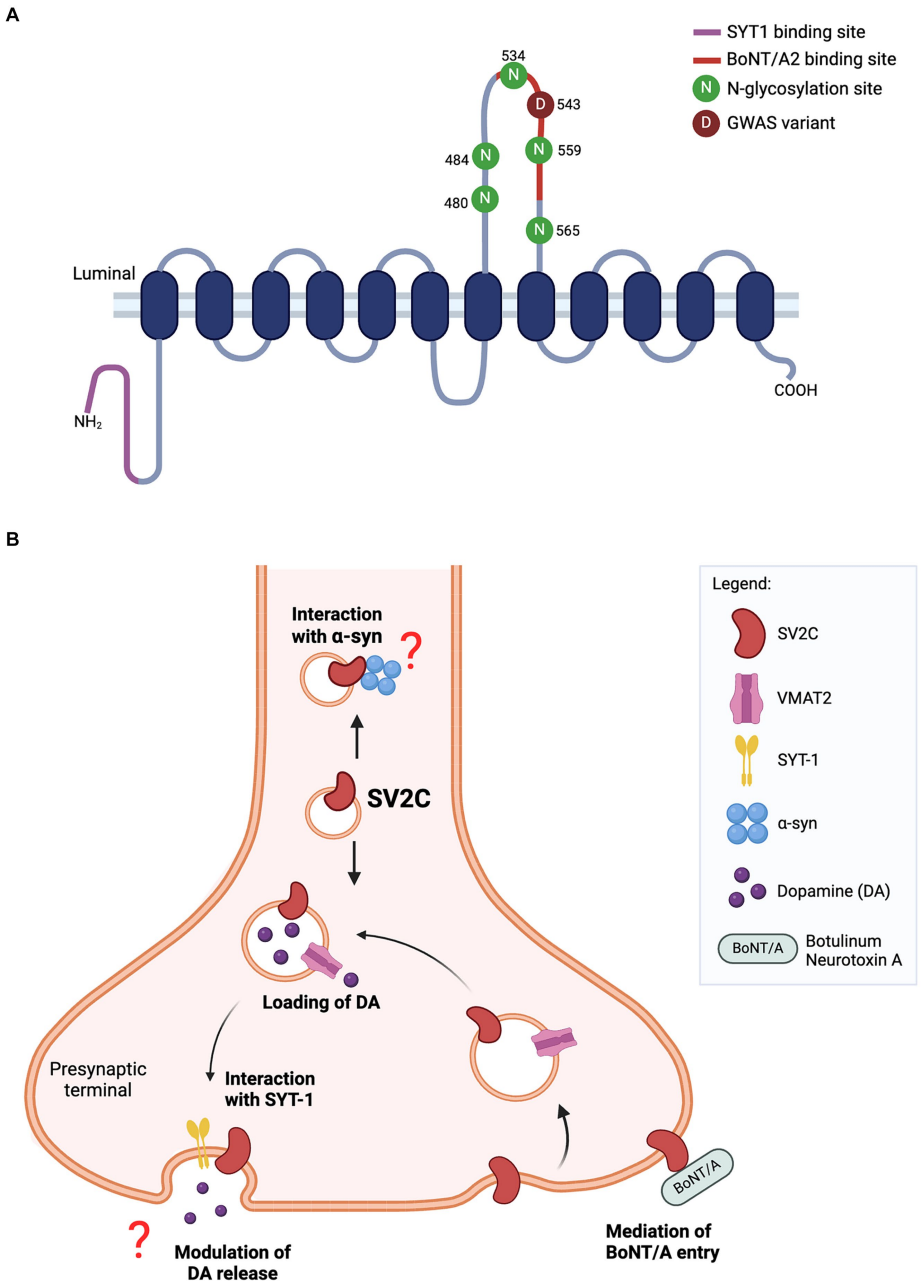
**(A)** Predicted structure of SV2C protein. The luminal domain contains five N-glycosylation sites while N-terminal tail harbors a binding site for synaptotamin-1 (SYT1). The missense variant (p.Asp543Asn) of SV2C reported in GWAS lies within the binding site for botulinum neurotoxin A2 (BoNT/A2). **(B)** Schematic of SV2C’s putative functions at the presynaptic terminal. SV2C is expressed on synaptic vesicles (SVs), where it may play a role in dopamine neurotransmission. SVs are loaded with dopamine through its transporter vesicular monoamine transporter 2 (VMAT2), and trafficked to the presynaptic membrane where dopamine is released through exocytosis. A key player mediating the exocytosis process is synaptotagmin-1 (SYT1) and SV2C has a unique binding site for SYT1. However, the functional significance of this interaction remains to be elucidated. Studies have reported the loss of SV2C decreased dopamine release, but the exact role of SV2C mediating this process is unclear. The highly-glycosylated luminal domain of SV2C faces the extracellular space and serves as a receptor for botulinum neurotoxin A (BoNT/A) entry into the neuron. Previous work also showed the direct interaction of SV2C with α-synuclein, details of which are yet to be fully understood (Schematics were built on BioRender).

In summary, the literature reviewed herein underscores the potential significance of SV2C in the pathogenesis of PD, particularly in the context of synaptic vesicle trafficking, dopamine neurotransmission and α-synuclein homeostasis (Figure 1). The potential pathways modulated by SV2C are beginning to be charted, yet the mechanisms by which SV2C exert its influence in PD pathology remain elusive. The precise stage at which SV2C influences the synaptic trafficking pathway is also unclear. Furthermore, the phenotypic impact of *SV2C* SNPs identified in genetic studies may differ from those observed in knockout models, indicating a call for comprehensive investigations in both the function of SV2C and the effects of these SNPs.

The majority of functional studies on SV2C have utilized animal models such as *Drosophila* and mice. While these models are indispensable for disease modeling, differences in genetic backgrounds between species, the physiological differences in the manifestation of PD symptoms and the disparity in complexity between human and mouse brains may limit the efficacy of animal models in fully comprehending the disease’s intricacies and translating potential therapeutic discoveries. This prompts the need for research within human cellular models and advances in stem cell technology, especially the use of patient-derived induced pluripotent stem cells, now provide us the unique opportunity to utilize midbrain dopaminergic-like neurons as an *in vitro* disease modeling tool (Kirkeby et al., 2012; Kriks et al., 2011). Elucidating the mechanisms of PD pathogenesis modulated by SV2C in both human and animal models will be crucial for a comprehensive understanding of the disease.

Genetic studies have pinpointed *SV2C* as a risk factor for PD, uncovering polymorphisms within the *SV2C* gene associated with PD risk and PD subtypes (Table 1). While GWAS has identified numerous risk loci, the effect sizes of these SNPs are often modest, posing challenges for functional validation (Blauwendraat et al., 2020). Yet, with the advent of precise gene-editing tools such as Clustered Regularly Interspaced Short Palindromic Repeats (CRISPR), we can now model genetic SNPs with remarkable accuracy in cellular models. Given that the rs246814 SNP is linked with a missense variant in the luminal domain of SV2C, with the latter potentially introducing new N-glycosylation, its functional validation is imperative. This will not only shed light on the role of SV2C in PD pathogenesis but may also position SV2C as a biomarker, potentially facilitating the stratification of PD subtypes for clinical trials and intervention. Furthermore, a recent structural analysis conducted by UCB (Union Chimique Belge) on SV2 ligands has unveiled new insights into the structural details for drug development aimed at targeting SV2C (Li et al., 2024). Together, these insights lay the groundwork for novel therapeutic strategies targeting SV2C in the management of PD.

**Table 1:** *SV2C* polymorphisms in PD.

SNP (rsid)	Type of SNP (gnomAD AF)	Association
rs30196	Intergenic variant (0.566)	Modifier of the effect of nicotine on PD risk (Hill-Burns et al., 2013)
		Associated with lower doses of levodopa (Altmann et al., 2016)
rs10214163	Intergenic variant (0.822)	Modifier of the effect of nicotine on PD risk (Hill-Burns et al., 2013)
rs246814	Intronic variant (0.0962)	Associated with increased PD risk in East Asian cohort (Foo et al., 2020)
		Associated with increased PD risk in European population (Grover et al., 2021)
		Associated with PD of mild subtype (Deng et al., 2022)
rs31244	Missense variant (0.103)	Tagged by SNP rs246814 (Foo et al., 2020)
rs1423099	5’ UTR variant (0.564)	Associated with lower incidents of adverse effects from dopamine treatment (Redenšek et al., 2019)
		Associated with increased PD risk in Han Chinese population (Tan et al., 2023)

Global allele frequencies (AF) were obtained from gnomAD v3.1.2.

As we continue to unravel the complexities of SV2C, the prospect of more precise diagnostic tools and targeted treatment approaches for PD, tailored to the molecular signature of individual patients, becomes increasingly tangible. In essence, advancing our understanding of SV2C’s role in PD not only promises to improve patient outcomes but also serves as a critical step toward mastering one of the most challenging neurodegenerative disorders of our time.

## Author contributions

CC: Data curation, Formal analysis, Investigation, Methodology, Visualization, Writing – original draft. KL: Conceptualization, Supervision, Writing – review & editing, Funding acquisition, Resources. JF: Conceptualization, Funding acquisition, Resources, Supervision, Writing – review & editing.
